# Atypical Manifestations of Syphilis: A 10-Year Retrospective Study

**DOI:** 10.3390/jcm13061603

**Published:** 2024-03-11

**Authors:** Giulia Ciccarese, Antonio Facciorusso, Mario Mastrolonardo, Astrid Herzum, Aurora Parodi, Francesco Drago

**Affiliations:** 1 Section of Dermatology, Department of Medical and Surgical Sciences, University of Foggia, Viale Pinto 1, 71122 Foggia, Italy; giulia.ciccarese@unifg.it (G.C.); mario.mastrolonardo@unifg.it (M.M.); 2 Gastroenterology Unit, Department of Medical and Surgical Sciences, University of Foggia, 00161 Foggia, Italy; antonio.facciorusso@unifg.it; 3 Dermatology Unit, IRCCS Giannina Gaslini, 16147 Genova, Italy; 4 Section of Dermatology, Department of Health Sciences, University of Genoa, IRCCS—Ospedale Policlinico San Martino, 16132 Genoa, Italy; aurorap@unige.it (A.P.); francescodrago007@gmail.com (F.D.)

**Keywords:** sexually transmitted diseases, sexual health, syphilis, coinfections, atypical chancre, reinfection, TPHA, VDRL, Follmann balanitis, psoriasiform syphilis, neurosyphilis

## Abstract

**Background**: The incidence of syphilis has increased in high-income countries in the past few decades, especially among men who have sex with men. In the present study, we aimed to analyze the correlations between atypical syphilis manifestations and the demographic, clinical, and laboratory features of patients and to review unusual presentations of syphilis reported in the literature. **Methods**: We conducted a retrospective analysis of 307 patients with syphilis diagnosed between 1 January 2013 and 31 October 2023 at the sexually transmitted infection (STI) centers of the University of Genoa and University of Foggia with both typical and atypical manifestations of disease. **Results**: In our series, atypical manifestations were detected in 25.8% of the patients, especially in the secondary stage of the disease. Lesions with annular morphology and lesions presenting as itchy erythematous scaly plaques with a psoriasiform appearance were the most common atypical presentations of secondary syphilis. A statistical analysis revealed that homosexual orientation, syphilis reinfection, and venereal disease research laboratory (VDRL) titers > 1:32 were correlated with atypical manifestations. **Conclusions**: Our study demonstrates that the spectrum of syphilis manifestations, in all the stages of the disease, is wide; atypical manifestations often pose diagnostic challenges, may delay the provision of appropriate treatment, and facilitate the spread of the infection.

## 1. Introduction

Syphilis is a sexually and vertically transmitted infection (STI) caused by the spirochete *Treponema pallidum* (TP), subspecies pallidum. Sexual transmission occurs during contact with an actively infected partner: the pathogens directly penetrate intact mucous membranes or abrasions in the skin, multiply locally, and shortly thereafter disseminate through lymphatics and the bloodstream. The natural clinical course of the disease typically encompasses three stages with asymptomatic latent periods between them [1].

The primary stage (primary syphilis, PS) is characterized by the appearance of a chancre at the site of TP inoculation, which is usually the anogenital area; the typical chancre is a 5–15 mm single, painless, indurated ulcer, with a raised edge and a clean base associated with regional lymphadenopathy. The signs of secondary syphilis (SS) present between 3 and 8 weeks after the appearance of the primary chancre and are protean. The patients may complain of low-grade fever, malaise, headaches, sore throat, conjunctivitis, and generalized arthralgia. Associated with these constitutional symptoms, the patients may have generalized lymphoadenopathy and, less commonly, enlargement of the spleen and liver. The typical rash of SS is a non-pruritic macular and/or papular exanthem, affecting the trunk and the extremities and often involving the palms and soles. Other typical signs that include mucous patches; non-scarring, non-erythematous alopecia of the scalp and beard often characterized by a moth-eaten appearance; lateral alopecia of the eyebrows; and condyloma lata (raised wart-like lesions on the moist areas of the body) are less common [1]. The eruption may last a few weeks to as long as several months. Latent syphilis is defined as an asymptomatic stage, with no clinical findings, in which the only evidence of the disease is reactive serologic testing. Latency may remain asymptomatic indefinitely, sometimes interrupted by a relapse of SS or progress to the tertiary stage.

Tertiary syphilis (TS) occurs in a third of patients in the absence of treatment [2] and may occur from 5 to 10 years or as long as 20 years after the primary infection, affecting mainly the skin, cardiovascular system (cardiovascular syphilis), or central nervous system (CNS) (neurosyphilis, NS). The cutaneous lesions are chronic, asymptomatic, asymmetric, slow-growing, and destructive and they may manifest as nodular, nodulo-ulcerative, or gummatous lesions. The main presentation during this stage involves the skin but gummas may appear in any organ such as bones, mucous membranes, and internal organs. The skin may ulcerate, resulting in crusted, scaly, nodulo-ulcerative lesions [1].

Gummas are painless granulomatous destructive nodules with little or no inflammatory reaction, occurring on the skin, or less often on the bones or internal organs. Initially, the nodule is firm, but due to progressive necrotic evolution, a gummy consistency develops [1].

Cardiovascular syphilis includes manifestations such as aortitis, aortic aneurysm, aortic valve disease, and coronary ostial occlusion. Involvement of the CNS can occur at any time in the course of syphilis: mild meningeal symptoms may happen in the early stages of syphilis; meningovascular syphilis, related to small artery inflammation in the brain/spinal cord, manifests as a stroke 5–10 years after infection; late neurological manifestations like tabes dorsalis, due to involvement of the posterior columns of the spinal cord, and general paresis, a progressive form of dementia, occur several decades after the infection [2,3]. These late manifestations, common in the pre-antibiotic era, are rare at present [1].

Prevalence of syphilis has increased in high-income countries in the past few decades, especially among men who have sex with men (MSM) [4].

Very recent studies on the epidemiology of STIs across the WHO European Region found a considerable increase in syphilis in European Union/European Economic Area (EU/EEA) countries, as opposed to decreasing trends in non-EU/EEA countries. More specifically, high rates (>8 per 100,000 population) were observed in northern European countries (e.g., Ireland, UK), western Europe (e.g., the Netherlands, Belgium), southern European countries (e.g., Spain, Malta), and some non-EU/EEA countries of eastern Europe (e.g., the Russian Federation). In these countries, syphilis notifications have substantially increased from 5 in 2012 to 7/100,000 in 2021, with the expansion of the population tested for syphilis and behavior changes potentially playing a role. On the contrary, the availability of STIs’ data in non-EU/EEA countries is more limited, with an inconsistent collection of information on modes of transmission, hindering comparability with EU/EEA data and conclusions about STI epidemics. However, data collected from national statistical reports in such non-EU/EEA countries indicate a steady syphilis decline [5].

Also considering the other continents, the highest incidence rate of syphilis from 1990 to 2019 occurred in central Sub-Saharan Africa (10·2 per 100,000 population) [6].

The resurgence of syphilis is closely associated with HIV infection and high-risk sexual behaviors (multiple sex partners, unprotected sexual intercourse, recent or current injection drug use, chemical sex) [7,8]. Interestingly, several studies have observed an increasing incidence of STIs, especially gonorrhea, chlamydia, and syphilis, after the beginning of pre-exposure prophylaxis (PrEP), a new preventive treatment for individuals at high risk for HIV infection. Indeed, knowledge of being on preventive treatment may reduce the individual risk perception of acquiring other STIs [9,10].

Like other chronic STIs, syphilis may impact the patient’s physical, psychological, and sexual health and negatively influence the quality of life [11]. In this context of syphilis worldwide resurgence, it is crucial not only to diagnose the form of typical syphilis but also to recognize the many atypical presentations of the disease.

We consider as atypical all manifestations that differ from the typical ones described above in morphology, number, localization, and associated symptoms. Regarding PS, atypical manifestations include chancres with atypical morphology (for example, dwarfed or giant in size, papulonodular without erosion, phagedenic or rhagadiform chancres), diffuse glans inflammation named “Follmann’s balanitis”, chancres localized in sites different from the anogenital area (extragenital chancres), chancres associated with local pain and multiple in number. In SS, the atypical morphology of the exanthem may be follicular, vesicular, pustular, nodular, ulcerative, corymbiform, psoriasiform, lichen-planus-like, or annular and the lesions may be pruritic. Moreover, in both PS and SS, atypical presentations with exclusive lymph node involvement or the simultaneous presence of PS and SS lesions (overlapping stages) have been described. Atypical manifestations of TS may be gummas in atypical locations or with atypical morphology, the rapid progression of aortitis, otosyphilis, and/or ocular syphilis. Neurosyphilis may be oligosymptomatic or completely asymptomatic [1,7,12].

In the present study, we described a case series of patients with syphilis presenting with both typical and atypical manifestations; we studied the correlations between atypical manifestations and the demographic, clinical, and laboratory features of patients; we also studied the current literature to review the unusual presentations of syphilis previously described.

## 2. Materials and Methods

We conducted a retrospective analysis of patients with syphilis diagnosed at the STI center of the University of Genoa and the STI center of the University of Foggia between 1 January 2013 and 31 October 2023. The physical examination of patients and clinical descriptions were performed by three dermatologists (G.C., F.D., and M.M.), experienced for decades in diagnosing and treating skin infections and STIs. The diagnosis of syphilis at any stage was based on clinical examination, patient’s history, and laboratory investigations. All patients clinically suspected for syphilis underwent serological screening to confirm the diagnosis: an enzyme-linked immunosorbent assay (ELISA) test for anti-*T. pallidum* IgM+IgG antibodies and, in the case of a positive result, a *Treponema pallidum* hemagglutination test (TPHA). In naive patients, the diagnosis was confirmed in the case of positivity of both tests. To monitor syphilis activity, non-treponemal tests (venereal disease research laboratory, VDRL) and ELISA tests for anti-*T. pallidum* IgM antibodies in serum were also performed both at the diagnosis and during follow-up. In patients with a previous history of syphilis, serology confirmed the clinical suspicion of reinfection when VDRL increased more than fourfold compared to the previous titer.

Each patient was also screened for HIV and hepatitis B and C virus infections. All the consenting patients were screened for seven sexually transmitted pathogens detected by a multiplex-polymerase chain reaction (PCR) analysis that was performed on biological samples collected through genital or anal swabs (based on the type of the reported sexual intercourses). These pathogens were Neisseria gonorrhoeae (NG), Chlamydia trachomatis (CT), Ureaplasma parvum (UP), Ureaplasma urealyticum (UU), Mycoplasma hominis (MH), Mycoplasma genitalium (MG), and Trichomonas vaginalis (TV). In addition, the consenting patients were also screened for genital, anal, and oropharyngeal high-risk (HR)-HPV infection through the collection of genital and/or anal samples with the Thin-Prep liquid-based cytology preparation system, as previously described [13,14].

Demographic, laboratory, and clinical data were saved on a computerized database. The following features were collected: gender, age at diagnosis, ethnicity, sexual orientation, stage of the disease (PS, SS, TS, NS), morphology, number, location, symptoms associated with the skin lesions, serology at diagnosis (TPHA, VDRL, and IgM antibodies), HIV status, coinfections other than HIV, and syphilis reinfection.

### Statistical Analysis

Patient characteristics were summarized using conventional statistics, using the mean and standard deviation for continuous variables and absolute frequencies and percentages for categorical data. An inferential analysis for correlation between baseline factors and occurrence of atypical syphilis was conducted through a uni/multivariate logistic regression analysis and results were expressed as odds ratios (ORs) and 95% confidence intervals (CIs). Significant variables in the univariate analysis were consistently entered into the multivariate model.

All statistical tests were 2-tailed, and differences were considered significant at a *p* value < 0.05.

The statistical analysis was run using the lrm package in R Statistical Software 3.0.2 (Foundation for Statistical Computing, Vienna, Austria) (Table 1).

## 3. Results

A total of 307 cases of syphilis diagnosed during the study period were included: 255 males and 52 females with a mean age of 39 years (ranging from 17 to 80 years) mainly of Caucasian ethnicity (91.2%). Sexual orientation was homosexual in 155 patients (50.4%), heterosexual in 145 (47.3%), and bisexual in 7 (2.3%). HIV status was positive for 13 patients (4%). The stage of disease was PS in 40 patients (13%), SS in 86 (29%), early latent in 76 (25%), late latent in 92 (30%), TS in 3 (1%), and NS in 7 cases (2%).

Patients with early and late latent syphilis were excluded from the analysis of atypical manifestations of syphilis since in such cases, there were no clinical manifestations of the disease [4].

The patients with PS, SS, TS, and NS diagnosed during the study period totaled 139. Their demographic, clinic, and laboratory features are described in Table 2.

Among these patients, most had SS (62%) and seven were HIV-seropositive (5%); the HIV diagnosis was performed at the same time as the syphilis diagnosis in two cases. Only one patient had a history of chronic HBV infection while no patients had HCV infection. Declared sexual orientation was homosexual in 72 patients (51%), heterosexual in 62 patients (45%), and bisexual in 5 (4%). TPHA and VDRL titers at diagnoses ranged from 1:80 to 1:10,240 and from negativity to 1:64, respectively. IgM antibodies against *T. pallidum* were tested on 98 patients and most of them (72%) were positive. One hundred and ten patients consented to be tested for other bacterial sexually transmitted pathogens and high-risk HPV infection: 48 tested positive for at least one bacterial infection (44%) and Mycoplasmataceae were the most commonly detected pathogens (40 patients, 37%); 47 patients tested positive for genital HR-HPV infection (34%), 15 for anal HR-HPV infection (11%), and 25 for oral HR-HPV infection (18%). In nine patients, syphilis was diagnosed as reinfection (7%), based both on the history and clinical and laboratory features.

The statistical analysis revealed that homosexual sexual orientation (*p* = 0.04), syphilis reinfection (*p* = 0.01), and VDRL titers > 1:32 (*p* = 0.02) were correlated with atypical manifestations of syphilis; conversely, the variables of sex, age, stage of the disease, HIV seropositivity, TPHA titers, *T. pallidum* IgM antibodies, and coinfections by other bacterial sexually transmitted pathogens and by HR-HPVs were not statistically correlated with atypical syphilis manifestations (*p* > 0.05).

### Atypical Syphilis Manifestations

Atypical presentations were observed in 36 of 139 patients with clinical signs of syphilis (25.8%). Of these, more than half (64%) were heterosexual males with a mean age of 42 years. HIV status was positive in two patients. Most atypical manifestations were observed in patients with SS followed by patients with PS. Interestingly, among the only 3 patients with TS in the present series, 2 (66%) had atypical skin manifestations and among the 10 patients with neurosyphilis, 3 (30%) had atypical presentations.

Regarding PS, atypical manifestations consisted of multiple genital chancres (three patients); Follmann balanitis (two patients); chancres in extragenital and extra-anal locations (two patients); and chancres with an extensive loss of substance (phagedenic or gangrenous chancres) in two cases. Atypical SS had many different manifestations: annular morphology of the lesions (five cases), lesions presenting as erythematous scaly plaques with a psoriasiform appearance (six patients), erythematous macule and papules of the hands and feet with scrotal eczema (two patients), anetoderma (one patient), overlapping stages of PS and SS (five cases, including the case with anetoderma), exclusive inguinal and cervical lymphadenopathy (two patients), necrotic and ulcerative lesions (three patient). Atypical cutaneous TS presented as lipoatrophic panniculitis in one case and as erythematous violaceous arciform plaques with peripheral ulceration on the back in another; in this latter case, the cerebrospinal fluid analysis was performed to rule out a CNS involvement and allowed us to diagnose asymptomatic neurosyphilis (the first patient with TS refused to perform the lumbar puncture). The two cases of atypical NS of this series were characterized by vertigo, headache, gait instability, generalized tonic–clonic seizures, dizziness, tinnitus, and leg numbness (Table 3).

## 4. Discussion

The old definition of syphilis as the “great imitator” due to its wide variety of clinical and dermatological manifestations is always factual. In 1978, Chapel found that less than half (42.7%) of patients with PS he diagnosed presented typical single, indurated chancres with regional lymphadenopathy, suggesting that the “classic” chancre may be the “atypical” lesions [15]. However, beyond many case report [16,17,18,19,20,21,22,23] and narrative review [24,25,26,27,28,29,30,31] syphilis manifestations, very few studies and/or case series including more than five cases with atypical syphilis manifestations have been published in the English literature during the past 20 years [32,33,34,35,36,37,38] (Table 4). Cases of atypical primary syphilis misdiagnosed as pharyngeal lymphoma, tongue cancer, and other types of oral squamous cell carcinomas have been described [39,40,41]; however, the secondary stage of the disease, given the possible involvement of the whole skin area and of other organs, is the one that is most prone to misdiagnosis like palmoplantar psoriasis, psoriasis vulgaris, erythema multiforme, cutaneous lymphoma, and granulomatous diseases of the skin (annular granuloma, sarcoidosis). Moreover, involvement of internal organs with signs and symptoms of osteomyelitis, nephrotic syndrome, retinitis, and others has been reported in secondary syphilis with or without typical cutaneous/mucosal involvement, further complicating the clinical picture [42,43,44,45,46,47]. In regards to neurosyphilis, a recent narrative review revealed a low level of clinical awareness of *T. pallidum* infection as a possible etiology of various psychiatric disorders including dementia, delirium, depression, mania, personality changes, and psychosis. All these disorders may represent atypical manifestations of neurosyphilis and considering that they are reversible with proper treatment, it should be imperative to implement routine screening for syphilis among psychiatric patients [48].

In our series, atypical manifestations were detected in 25.8% of the patients with clinical signs of syphilis.

The rate of atypical early syphilis is similar to that observed by Arando M. et al. (31%); however, they observed multiple chancres and overlapping of PS and SS more often in HIV-positive patients [37]; conversely, in our study, atypical manifestations were not correlated with HIV infection or with other STIs.

**Figure 1:**
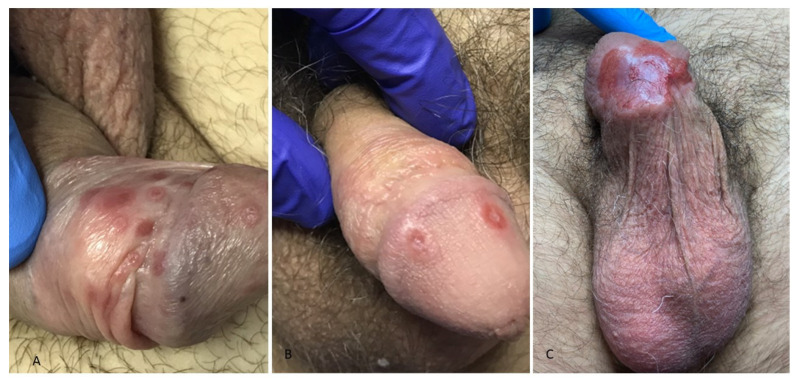
Atypical manifestations of primary syphilis: (**A**) Follmann balanitis characterized by erythematous, concentric, rosette-like lesions on the balanopreputial sulcus, glans, and foreskin; (**B**) two identical chancres of the glans; (**C**) phagedenic chancre: chancre manifesting as an erythematous, infiltrated, and ulcerated plaque with an extensive loss of tissue involving the balanopreputial sulcus and the glans; superinfection by *S. agalactiae* was detected through a cutaneous swab on the lesion.

Two other studies based on a conspicuous number of patients with PS detected higher frequencies of atypical chancres (half of the cases) [33,35] compared to our series (21%) and described a wider variability of morphological variants: papulonodular chancres without erosion, erosive chancres without infiltration, ragadiform, herpetiform, and diphteroid chancres reported by Ramoni S. et al. [33] were never observed in our patients; conversely, gangrenous chancres and Follmann’s balanitis were as common [33] as in our series. More specifically, Follmann balanitis, characterized by erythematous, concentric, rosette-like lesions (Figure 1A), found in one of our patients, was never described previously.

In our study, extragenital chancres were exclusively located in the oral cavity whereas they were mainly found in the anal region, especially in MSM, by other authors [33,35,37]. The simultaneous presence of multiple chancres, observed in three cases of PS (7.5%) in our series (Figure 1B), was more commonly described in other reports [15,33,34] independently from HIV status. Multiple chancres may result from multiple *T. pallidum* inoculations during the same sexual intercourse or from autoinoculation beginning from the first chancre [15]. Phagedenic chancres, observed in two cases of our series (5% of PS, Figure 1C), were reported with the same frequency by Ramoni S. et al. [33].

The phagedenic chancres have been historically associated with pyogenic superinfections (Streptococcus agalactiae in one of our cases), which cause an extensive loss of substance; in other instances, immunosuppression due to HIV infection and/or alcoholism may cause the loss of local cellular immunity, favoring the massive local diffusion of the *T. pallidum* infection [29,38]. None of our patients with phagedenic chancres were HIV-seropositive, but one was a heavy drinker and the other used soft drugs.

**Figure 2:**
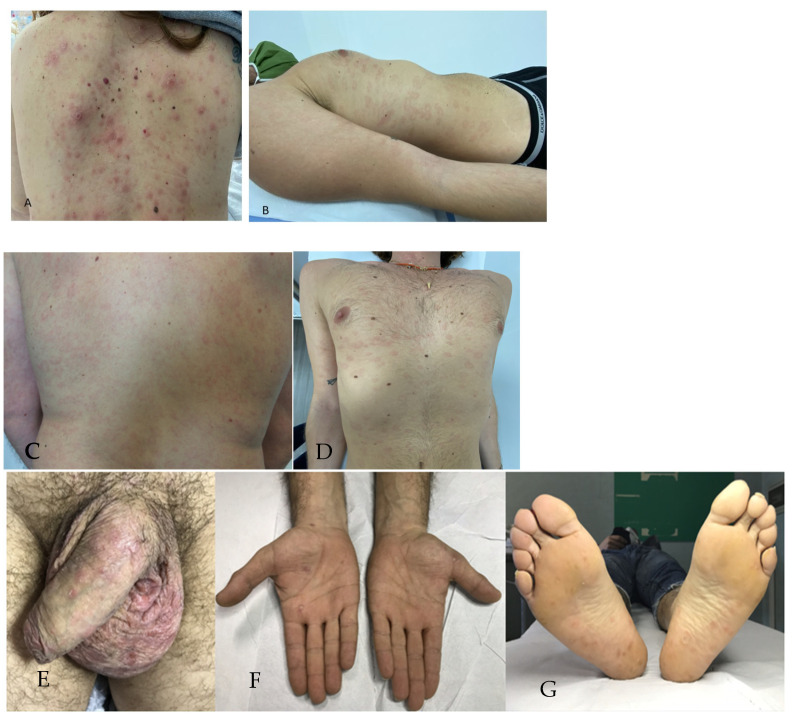
Atypical manifestations of secondary syphilis: (**A**) psoriasiform secondary syphilis—slightly infiltrated papules and plaques covered by thick adherent scales of the back; (**B**–**D**) annular secondary syphilis: erythematous slightly infiltrated macules with annular shapes on the trunk, upper limbs, and back; (**E**–**G**) scrotal and shaft penis eczema in a patient with typical lesions of secondary syphilis on the palms and soles.

Concerning SS, the rate of atypical manifestations found in our series (25.8%) was similar to that reported by Arando M. et al. (31%) [37]. The most common atypical presentation in our patients was the psoriasiform appearance, characterized by infiltrated plaques over the trunk covered by thick adherent scales, mimicking eruptive psoriasis (Figure 2A). These rashes, especially when associated with arthralgia in patients with personal or family history of psoriasis, may be easily misdiagnosed. In our series, only one of the six patients with psoriasiform syphilis had a family history of psoriasis; however, the simultaneous diagnosis of PS in the partner and complete resolution after treatment confirmed the diagnosis. Psoriasiform SS has been described both in immunocompetent patients and in people living with HIV [29,38,49]. This clinical aspect corresponds histologically to a dense mononuclear infiltrate composed of lymphocytes, macrophages, plasma cells, neutrophils, and eosinophils in variable proportions, extending to the deep dermis [29].

Annular syphilis was the second most common variant of atypical SS in our series: lesions were erythematous slightly infiltrated macules with annular shapes on the trunk and limbs (Figure 2B) and in one case, only on the palms [20]. In our series, as previously described [20], this variant was common in patients who experienced syphilis reinfection (three out of five patients). In the literature, annular SS morphology is variable, with lesions ranging from slightly scaling papules to verrucous papules/plaques that can affect the scalp (cause scarring alopecia), trunk, perioral-, perianal-, and genital regions [25]. There are several possible mechanisms responsible for the annular shape: lesions simply form at a site and then spread radially; a lesion can have a centrifugal spread by extension along a plane in the skin; or an inflammatory process may extend along a vessel and, because the vessels are arranged in a grid-like network, the clinical result is a figurate lesion [50].

Scrotal eczema, associated with the typical manifestation of erythematous macules/papules of the palms and soles, was another atypical manifestation of SS in our series; to the best of our knowledge, this type of lesion was previously reported only in four cases, two of which were HIV-seropositive [51,52,53]. Although in our patients with scrotal eczema histopathology and immunohistochemistry for a polyclonal antibody against *T. pallidum* were not performed, the concomitant diagnosis of syphilis in the sexual partner in one case and personal history of several unprotected sexual intercourses in the other, together with the complete resolution of eczema after specific antibiotic therapy, suggested that these lesions were atypical manifestations of syphilis.

Concerning anetoderma, this condition of loss of elastic tissue in the dermis may be idiopathic or it may happen in the context of infectious, autoimmune, or neoplastic diseases; indeed, SS has been reported as one of its common causes. Two distinct clinical subtypes of syphilitic anetoderma have been described: one characterized by multiple atrophic lesions in a widespread, pityriasiform distribution, and the other by fewer, well-defined, convex, atrophic lesions, as in our patient [21,54,55].

**Figure 3:**
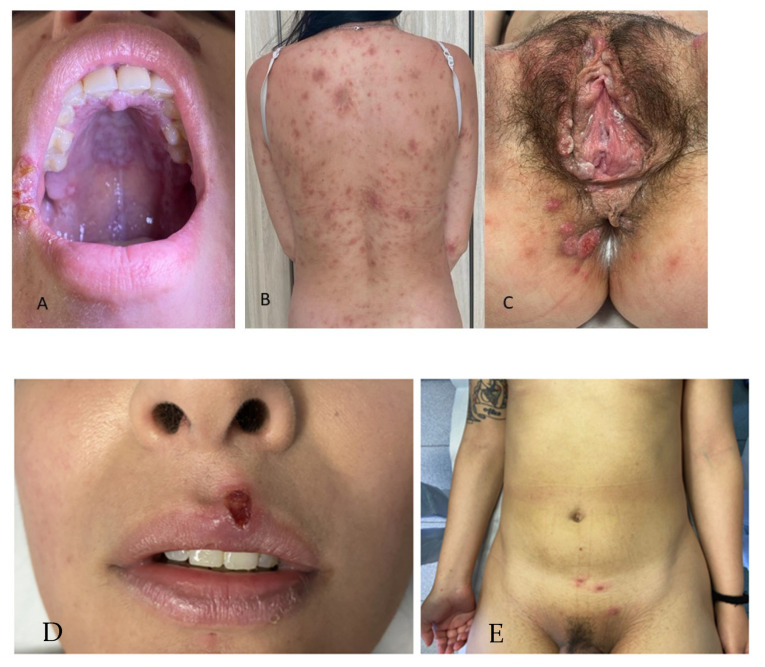
Overlapping stages of primary and secondary syphilis: (**A**–**C**) concomitant presence of the right labial commissure chancre, psoriasiform exanthem of the trunk, and genital mucosal patches; (**D**,**E**) chancre of the upper lip, erythematous papules of the arm, and papulonodular lesions of the pubis.

Overlapping stages of PS and SS were quite common in our series (14% of the atypical manifestations, Figure 3) and always occurred in HIV-seronegative patients, differently from other previous studies [56,57]. It is difficult to establish whether these overlapping stages of syphilis represented a slower-than-normal healing of the lesions of PS or an accelerated progression to SS.

Two of our patients had inguinal and cervical lymphadenopathy as the exclusive clinical signs of syphilis: the positive serology for *T. pallidum* infection and the negative serology for HIV, HBV, and HCV infections and for active infections by Epstein Barr virus, Citomegalovirus, Bartonella henselae, and Toxoplasma gondii, together with the complete resolution after specific antibiotic therapy, allowed us to diagnose these lymphadenopathies as luetic. The absence of any cutaneous/mucosal lesions in these cases could be explained by the peculiarity of the chancre of PS, which classically presents as a painless and small nodule that spontaneously heals without sequelae and therefore can go unnoticed. *T. pallidum* is typically responsible for persistent, indolent inguinal lymph node enlargement during PS, whereas in later stages, lymphadenopathy is usually generalized and associated with systemic symptoms [1]. However, when lymphadenopathy remains confined to one region, as in our patients, syphilis should be suspected regardless of the absence of skin lesions or positive early serology results [12]. Indeed, non-treponemal tests can be falsely negative due to the prozone phenomenon [58].

**Figure 4:**
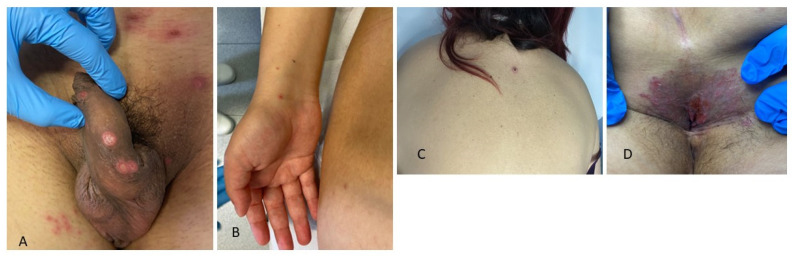
(**A**,**B**) Malignant syphilis: multiple necrotic ulcerated papules in the genital area associated with erythematous macules and papules of the arms (patient 1); (**C**,**D**) isolated necrotic lesion of the back associated with mucosal patches of the perianal region (patient 2).

Necrotic ulcerative lesions represent a rare, severe manifestation of SS (malignant syphilis) that has been described largely in MSM with HIV coinfection and, recently, also in association with immunosuppressive treatment [59]; however, it can also occur in immunocompetent individuals, as in our patients. Malignant syphilis is characterized by prodromal symptoms (fever, chills, headache, weight loss, and generalized lymphadenopathy), followed by the appearance of multiple, irregularly distributed lesions that are clinically similar to primary chancres. Lesions with different morphologies, including papules, nodules, pustules, and ulcerations covered with brown rupioid crusts, may be present. Moreover, this variant can present with neurological symptoms, and spirochetes may be isolated in cerebrospinal fluid. In our three female patients with malignant syphilis, multiple necrotic ulcerated papules were visible on the genital area in two cases while there were a few lesions on the trunk in the other (Figure 4A–D); neurological symptoms were absent. Malignant syphilis is not a malignancy but it was given this name in 1859 based on its severe clinical features. Several pathogenetic theories have been postulated to explain its development. First, it could be related to an abnormally elevated immune response: studies of malignant syphilis tissues have demonstrated very few organisms and an excessive inflammatory infiltrate; secondly, interaction with HIV or another cause of immunosuppression may result in this particular aspect; the last theory suggests that malignant syphilis results from strains of *T. pallidum* with increased virulence [60,61]. Interestingly, the fact that most HIV-seropositive patients with malignant syphilis do not have a severely decreased CD4+ count may suggest that the pathogenesis of malignant syphilis is not related to a quantitative deficit in the immune system but rather to a qualitative immune dysfunction [60].

A recent retrospective review of NS and TS cases reported in Alberta from 1973 to March 2017 found that NS cases (251 cases) continue to occur in the context of cycling syphilis outbreaks while cardiovascular syphilis cases were rare (three patients) and TS (or gummatous syphilis) was absent [61]. The most common manifestations of NS were ocular involvement (more likely in early NS) and cognitive impairment; patients with early NS were more likely to be younger, Caucasian, HIV-seropositive, and reporting same-sex partners [62]. Conversely, in our literature review on the clinical presentation of NS from 1 January 2010 to 31 December 2014, we found that NS presented in most patients as cognitive impairment and psychiatric symptoms (49%) followed by ocular or auditory symptoms (22%); HIV infection was detected only in a minority of patients (10%), 26. This study suggested that oligosymptomatic and/or atypical forms of NS are becoming commoner than the classical early and late manifestations (syphilitic meningitis, meningovascular syphilis, general paresis, tabes dorsalis, and gummas) [63]: in front of suggestive symptoms, NS must be suspected and CSF examined, regardless of whether the conventional treatment has been performed. Indeed, the possible existence of particularly neuroinvasive *T. pallidum* strains and their possible phenotypic resistance to penicillin in the host environment should not be neglected [3]. It is well known that *T. pallidum* is sensitive to the treatment only when it is in replication (every 33 h); therefore, when its replication is slower/absent, as in the late syphilis stages, penicillin may be ineffective [64]. Although it is still considered the treatment of choice for syphilis [8], several reports found that penicillin may be inadequate in preventing all syphilis late complications such as neurosyphilis [3,65,66,67,68,69]. In addition, allergic reactions and poor drug tolerance represent challenging problems for the use of this antibiotic; therefore, the search for new therapeutic strategies has attracted the attention of many researchers [69,70]. A recent network meta-analysis on the efficacy and safety of treatments for different stages of syphilis showed that ceftriaxone was more effective than penicillin in treating syphilis after follow-up of 6 months, but more clinical studies are needed to support its use as an alternative to penicillin [70].

Nowadays, TS is a rare disease, mainly because of the widespread use of antibiotics for concomitant infections [1]; therefore, case series describing its possible atypical manifestations are lacking. However, several case reports showed the multifaceted presentation of this stage of the disease: in the past two years, cases of atypical TS manifested as cutaneous annular plaques of the arms [71], palpable testicular masses [72], and crusting nodules of the scalp [73] have been documented. TS often represents a diagnostic challenge due to negative non-treponemal tests in up to 30% of cases and to the frequent lack of identifiable spirochetes on histopathology [74].

There is a correlation between atypical syphilis and demographic, clinic, and laboratory features.

In our series, atypical manifestations of syphilis were statistically correlated with homosexual orientation, syphilis reinfection, and VDRL titers > 1:32.

Like in our study, Ramoni et al. found that chancres with atypical locations (extragenital site) were more common in MSM than in heterosexual men [33]. Conversely, no significant differences were observed in terms of the number of lesions (multiple vs. single), chancre morphology, or serology at diagnosis (negative vs. positive) between MSM and heterosexual men as well as between HIV-seropositive and HIV-seronegative patients [33]. The association between extragenital chancre location and homosexual behavior [35,75,76] is likely related to oral–genital and oral–anal intercourses, the most frequently reported sexual practices among MSM [77].

Regarding syphilis reinfection, it has recently been reported that previous syphilis may attenuate the clinical manifestations and the laboratory parameters of a new *T. pallidum* infection, which occurs with less severe, and therefore atypical, skin manifestations or presents as latent syphilis [78]. Marra et al. also showed that the odds of the detection of *T. pallidum* DNA and rRNA in blood and CSF at the index episode were lower in individuals with previous syphilis compared with those without previous syphilis [79]. Whether this finding reflects the acquired immune responses is not completely clear.

The concept that previous syphilis can confer protection from reinfection is supported also by prior observations in rabbits and human prisoners [79]. This concept would imply that an effective syphilis vaccine may be possible; however, this goal is still far from being achieved. Anyway, the finding that previous syphilis may attenuate the clinical manifestation of a new *T. pallidum* infection has important implications for public health and clinical practice, supporting the recommendation to regularly screen the high-risk individuals for syphilis, regardless of symptoms.

In regards to non-treponemal test titers, previous studies reported that HIV-seropositive patients with syphilis more commonly have unusually high titers of serological tests or false-negative results (prozone phenomenon), compared to HIV-seronegative patients [57]. In our series, the correlation between VDRL titers > 1:32 and atypical syphilis manifestations occurs regardless of the HIV status of the patients. The reason why this correlation was found only with non-treponemal tests (and not with treponemal tests) remains unclear.

## 5. Conclusions

Our study demonstrates that the spectrum of syphilis manifestations, in all stages of disease, is wide; atypical manifestations often pose diagnostic challenges, may delay appropriate treatment, and facilitate the spread of infection.

Given the increase in syphilis incidence in recent years, the probability that dermatologists may encounter atypical disease manifestations is increasing, as well. Therefore, clinicians should maintain a high level of clinical suspicion and be able to recognize the atypical presentation of syphilis at any stage.

## Figures and Tables

**Table 1:** Logistic regression analysis of predictors for atypical manifestations of syphilis.

	Univariate Analysis		Multivariate Analysis	
Variables	Odds Ratio (CI 95%)	*p*-Value	Odds Ratio (CI 95%)	*p*-Value
Sex (reference, female)	0.40 (0.15–1.09)	0.07		
Race (reference, Latin)	4.15 (0.61–1.86)	0.89		
Age	1.62 (0.87–3.93)	0.07		
Sexual orientation (reference, homosexual/bisexual)	0.34 (0.12–0.96)	0.04	0.45 (0.24–0.91)	0.03
Diagnosis (reference, PS)	SS 1.9 (0.54–6.58)	0.31		
	TS-NS 4.75 (0.82–27.49)	0.08		
HIV status (reference, negative)	1.11 (0.12–9.69)	0.92		
Reinfection (reference, no)	16.76 (1.83–153.48)	0.01	18 (1.33–172.11)	0.02
TPHA (reference, ≥5120)	0.87 (0.35–2.15)	0.01		
VDRL (reference, ≤32)	2.18 (1.08–5.38)	0.03	2.11 (1.13–5.15)	0.02
*T. pallidum* IgM antibodies (reference, negative)	1.33 (0.52–6.78)	0.97		
Coinfections by other sexually transmitted bacteria and by HPV (reference, negative)	1.17 (0.38–3.59)	0.77		
HBV/HCV coinfections (reference, negative)	0.50 (0.02–10.87)	0.66		

Abbreviations: CI 95%, confidence interval 95%; PS, primary syphilis; SS, secondary syphilis; TS, tertiary syphilis; NS, neurosyphilis; HIV, human immunodeficiency virus; VDRL, venereal disease research laboratory; TPHA, *Treponema pallidum* hemagglutination assay; HBV, hepatitis B virus; HCV, hepatitis C virus; HPV, human papillomavirus.

**Table 2:** Demographic, clinic, and laboratory features of the patients with PS, SS, TS, and NS.

Syphilis with Clinical Signs: Primary, Secondary, and Tertiary Syphilis and Neurosyphilis		
	Total n° = 139	Atypical Syphilis n° = 36
Gender		
Male	111 (80%)	25 (69%)
Female	28 (20%)	11 (31%)
Mean age at diagnosis, year (range)	38 (17–75)	42 (23–67)
Ethnicity		
Caucasian	129 (93%)	36 (100%)
Latin	10 (7%)	0
Sexual orientation		
Heterosexual	62 (45%)	23 (64%)
Homosexual	72 (51%)	12 (33%)
Bisexual	5 (4%)	1 (3%)
Stage of the disease		
Primary	42 (30%)	9 (25% of the atypical syphilis, 21% of the primary syphilis)
Secondary	84 (60%)	22 (61% of the atypical syphilis, 26% of the secondary syphilis)
Tertiary	3 (2%)	2 (5% of the atypical syphilis, 66% of the tertiary syphilis)
Neurosyphilis	10 (7%)	3 (8% of the atypical syphilis, 30% of the neurosyphilis)
HIV status		
Positive	7 (5%)	2 (5%)
Negative	132 (95%)	34 (95%)
Serology at diagnosis		
TPHA mean titers (range)	5120 (80–10,240)	5120 (640–10,240)
VDRL mean titers (range)	32 (neg-64)	32 (neg-64)
Positive IgM anti-*T. pallidum* (tested on 98 patients)	100 (72%)	26
Bacterial sexually transmitted coinfections (tested on 110 patients)		
Patients positive for at least one pathogen	48 (44.4%)	10 (28%)
Genital/anal *C. trachomatis* infections	4 (3.6%)	2 (5%)
Genital/anal *N. gonorrhoeae* infections	2 (1.8%)	0
Genital/anal infections by Micoplasmataceae	40 (37.2%)	10 (28%)
Genital *T. vaginalis* infections	2 (1.8%)	0
Other concurrent STIs		
Genital warts	2 (1.5%)	0
Genital high-risk HPV infection (tested on 110 patients)	47 (34%)	0
Anal high-risk HPV infection (tested on 110 patients)	15 (11%)	2 (5%)
Oral high-risk HPV infection (tested on 110 patients)	25 (18%)	3 (8%)
Chronic HBV infection	1 (0.7%)	0
Herpes genitalis	1 (0.7%)	1 (3%)
Monkeypox	1 (0.7%)	0
Syphilis reinfection	9 (6.9%)	5 (14%)
Atypical manifestations	36 (25.8%)	

Abbreviations: PS, primary syphilis; SS, secondary syphilis; TS, tertiary syphilis; NS, neurosyphilis; HIV, human immunodeficiency virus; HPV, human papillomavirus; HBV, hepatitis B virus; TPHA, *Treponema pallidum* hemagglutination assay; VDRL, venereal disease research laboratory.

**Table 3:** Demographic, clinic, and laboratory features of the patients with atypical manifestations of syphilis.

Atypical Syphilis									
Patient n°	Sex	Age at Diagnosis	Sexual Orientation	Stage of Disease	Clinical Features	HIV Status	Reinfection	VDRL Titer	TPHA Titers
1	M	32	homosexual	PS	oropharyngeal chancre	neg	no	64	10,240
2	M	44	homosexual	PS	multiple chancres	neg	no	64	2560
3	M	47	heterosexual	PS	phagedenic chancre	neg	no	neg	640
4	M	53	heterosexual	PS	phagedenic chancre + multiple chancres	neg	no	2	2560
5	M	39	heterosexual	PS	multiple chancres	neg	no	32	1280
6	M	47	homosexual	PS	oropharyngeal chancre	neg	no	16	2560
7	M	31	homosexual	PS	multiple chancres	neg	no	64	1280
8	M	53	heterosexual	PS	Follman balanitis: glans lesions with rosette-like appearance	neg	no	32	10,240
9	M	23	heterosexual	PS	Follman balanitis	neg	no	16	5120
10	M	31	homosexual	SS	exclusive inguinal and cervical lymphadenopathy	neg	yes	neg	10,240
11	F	43	heterosexual	SS	erythematous–scaly plaques on the lower legs (psoriasiform syphilis)	neg	no	16	10,240
12	M	28	homosexual	SS	annular lesions of the trunk and arms	neg	yes	16	1280
13	M	54	heterosexual	SS	annular lesions of the palms	neg	yes	32	10,240
14	M	28	homosexual	SS	annular lesions of the trunk	pos	no	16	5120
15	M	47	heterosexual	SS	erythematous macules and papules of the palms and soles and scrotal eczematous plaques	neg	yes	32	10,240
16	M	26	homosexual	SS	overlapping stages: Follman balanitis and anetoderma	neg	no	2	1280
17	F	47	heterosexual	SS	few diffuse necrotic ulcerative lesions (lues maligna)	neg	no	16	5120
18	F	34	bisexual	SS	overlapping stages: chancre of the upper lip, papules of the arms, multiple necrotic ulcerative lesions (lues maligna) of the genitalia	neg	no	32	5120
19	F	27	heterosexual	SS	exclusive inguinal and cervical lymphadenopathy	neg	no	64	10,240
20	F	31	heterosexual	SS	itchy erythematous scaly papules and plaques on the trunk (psoriasiform syphilis)	neg	no	32	10,240
21	M	41	homosexual	SS	erythematous–scaly plaques on the trunk and legs (psoriasiform syphilis)	neg	no	8	2560
22	M	40	homosexual	SS	diffuse erythematous–scaly plaques (psoriasiform syphilis)	neg	no	16	2560
23	F	38	heterosexual	SS	diffuse erythematous–scaly plaques (psoriasiform syphilis)	neg	no	32	1280
24	M	30	homosexual	SS	annular lesions of the trunk	neg	yes	16	5120
25	M	35	homosexual	SS	annular lesions of the trunk	pos	no	neg	10,240
26	F	37	heterosexual	SS	overlapping stages: chancre of the labia majora, papules of the trunk and arms	neg	no	16	10,240
27	M	29	heterosexual	SS	overlapping stages: Follman balanitis and maculo-papular exanthem	neg	no	16	5120
28	M	50	heterosexual	SS	erythematous papules of the palms and soles and scrotal eczematous plaques	neg	no	8	10,240
29	F	35	heterosexual	SS	few diffuse necrotic ulcerative lesions (lues maligna)	neg	no	8	2560
30	F	30	heterosexual	SS	overlapping stages: chancre of the upper lip, macules and papules of the trunk and arms	neg	no	32	10,240
31	M	40	heterosexual	SS	itchy erythematous–scaly papules and plaques on the trunk (psoriasiform syphilis)	neg	no	16	1280
32	F	67	heterosexual	TS	panniculitis	neg	no	16	1280
33	F	59	heterosexual	TS and NS	erythematous violaceous arciform plaque with peripheral ulceration	neg	no	64	10,240
34	M	59	heterosexual	NS	vertigo, headache, gait instability, generalized tonic–clonic seizures	neg	no	64	1280
35	M	64	heterosexual	NS	dizziness, tinnitus, fainting episodes presented with headache, gait instability, and leg numbness	neg	no	64	640
36	M	61	heterosexual	NS	vertigo, headache, gait instability	neg	no	32	640

**Table 4:** Studies on the atypical syphilis manifestations published in the past 20 years.

Case Series of Atypical Syphilis Manifestations Published in the Past 20 Years				
First Author	Year	Stage of Disease Studied	N° of Studied Patients	Rate of Atypical Syphilis
Towns JM. et al. [35]	2016	primary	183	49%
Arando M. et al. [37]	2019	primary, secondary, and early latent	274	31%
Skalnaya A. et al. [32]	2019	neurosyphilis	6	50%
Sardinha JC. et al. [38]	2020	primary and secondary	19	100%
Larsen C. et al. [34]	2020	primary	12	100%
Ramoni S. et al. [33]	2021	primary	244	50.4%
Gong HZ. et al. [36]	2021	primary	27	100%
Present study	2023	all	139	26%

## Data Availability

Data available upon reasonable request.
